# Bibliographical Notices

**Published:** 1855-10

**Authors:** 


### BIBLIOGRAPHICAL NOTICES.

*Yellow Fever, considered in its Historical, Pathological, Etiological, and Therapeutical Relations: Including a sketch of the Disease as it has occurred in Philadelphia from the year* 1699 *to* 1853 ; *with an examination of the connection between it and the Fevers known under the same name in other parts of Temperate as well as in Tropical Regions.* By R. La Roche, M. D., &c., &c. Two volumes, pp. 615 and 813. 8vo., with a Bibliography. pp. 45.

Two large volumes, containing nearly fifteen hundred closely printed pages on the subject of Yellow Fever alone. We can readily conceive what a sensation will be produced by the mere announcement of this fact, on the entire medical profession, the members of which will be at once divided, on the occasion, into two great parties, each containing its subdivisions. We do not advert now to the differences of opinion on the doctrines involved in a discussion of the origin, spread and treatment of yellow fever, which are so thoroughly investigated in its pages. Our reference is to the different feelings with which it will be received by, on the one part, the many who scarcely read at all, added to the goodly number of those who think they do their duty when they read a paper or two in each successive number of some medical journal; and, on the other part, by conscientious students and observers who are intent on learning, as far as possible, through every known channel, and from every reliable quarter, all the phenomena of diseases, and all the means ever used for their prevention and cure, in order that they may be prepared for any emergency, whether it be in the shape of a new rebellious outbreak of endemia, or of a direful siege by pestilential epidemia, or the combined assaults of both. The first mentioned classes of the brethren will, we fear, be disposed to look on Dr. La Roche's work in the light of an inconvenient reminder of a duty, which, wanting inclination to discharge, they may try to evade, under the plea of its being unnecessarily voluminous. Nor is the plea without weight when made by those who hardly ever venture on the perusal of a volume of even the most diminutive size. By the studious, the inquiring, the progressive and improving members of the profession (do they constitute a majority?) this work will be received with gratitude as a real boon, and read with avidity, and afterwards treasured up for ready reference, as an encyclopædia, in which all the facts, explanations, and elucidations bearing on the whole subject of yellow fever, will be found under appropriate heads; at the same time that these are presented in the several relations and connections which belong to a systematic treatise.

The reasons for an extended history of the kind, are, 1, the importance of the subject; 2, the amount and value of the records from which the materials are obtained for its composition. On the first of these points there can scarcely be, in relation to the present work, a difference of opinion. A disease which, at various times, has assailed all the great marts of commerce in the United States, from Boston to New Orleans, and inflicted on their inhabitants a greater loss of life, and incomparably greater gloom and distress, and interruptions to social and business intercourse, than would have ensued from siege and blockade, must excite the attention of the most indifferent and rouse the sympathies of the most apathetic. It is, indeed, a fit and a great theme for history, viewed as a methodical record of startling and fearful events, mingled with grave reflections, and set off by vivid pictures of human nature in its most contrasted features; here of intense selfishness and cowardice; there of devotion to others at a sacrifice of personal comfort, health and life itself, in a more heroic spirit than would prompt the bravest soldier to join the forlorn hope and advance to "the imminent deadly breach."

In other lands on this Continent, and in some of the chief cities of Southern Europe, yellow fever has, also, proved a scourge, more severe and destructive in some instances than it has been in our country; and in them, also, death's doings are invested with an importance which fits them for the page of history.

Of the number and copiousness of the writings on yellow fever, in the shape of separate volumes, essays, and papers in Medical Journals and the Transactions of Societies, Official Reports, &c., in different languages, constituting rich materials for a full history, the most superficial reader cannot now be ignorant, thanks to the patient researches of Dr. La Roche. In a Bibliography of forty-five pages, prefixed to the first volume, we read the titles of nearly a thousand (987) different productions of the pen, more or less descriptive of or bearing on the subject of this fever. We may add, that, if we except a hundred or so of this long catalogue, all the works in it have passed under the inspection of the author of the volumes now before us, and been turned by him to suitable account. The larger number of these, "*Memoires pour servir à l'histoire*" are in the English and French languages; some in Spanish and Italian, and a few in German and Latin. Every page of his own work bears proof of the frequency and extent of his references to these commemorative writings of his predecessors, and of not a few of his contemporaries, while often repeating their own words on the occasion.

It would be presumptuous to assert that Dr. La Roche is the first to write a methodical Treatise on Yellow Fever, or that all the topics of which he treats have not been examined separately, and nearly under every aspect, by some one writer or another, and often by many writers. We could quote passages from them of more vivid description and easier flow of narrative, and of scenes of touching interest more dramatically told. But, in vain shall we look around us with the expectation of finding, in any language, the medical history of yellow fever in all its amplitude of outline and minuteness of detail, brought down to the present day, and written with the severity of logic, and the illustrative aids of natural philosophy, chemistry, morbid anatomy and micrology, and the statistical tests of the relative extent of the causes, the mortality and the results of the treatment of this fever, which impart such distinctive features to these volumes. They constitute, in every sense, a great work, whether we regard its size, the labor spent in its preparation, or the learning in collecting, and the assiduity and method in the distribution and arrangement of the materials.

The author's mind is essentially analytical, and hence he loves to study each element of the subject under examination, separately, rather than in its contingent aspects and incidental relations. In this, if restricted to an alternative, he is undoubtedly correct; but we might sometimes wish, that after the separate parts were examined and thoroughly scrutinized, they had been more frequently grouped, so as to exhibit the entire picture, with the incidental yet characteristic touches which increase its effects. To this it may be replied, that he writes the internal, the material history of yellow fever, and leaves its psychological, ethical and social bearings and results to other pens who may choose to describe them. He would not have satisfied either himself or his readers by the few bold and graphic touches with which Thucydides places before us the plague that ravaged Athens, in the second year of the Pelopennesian war, nor by the minute personal descriptions and occasional dialogue, assuming at times a dramatic character, which mark De Foe's History of the Great Plague of London in 1665. Still less would he care to imitate Lamartine, even if he possessed the poetical fervor and rich fancy of this writer, by the production of a series of brilliant pictures in which chronology is forgotten and accuracy too often sacrificed to effect.

In our notice of the present work we can only hope, after having stated our impressions of its meritorious character and of its opportune appearance at this time, to introduce some evidences of the author's patient and laborious method of investigation, and the heads of the prominent topics of which he treats. In his preface he gives the reasons which influence him in writing the work, and some explanations of its general plan. We should prefer seeing the explanatory part transferred to the beginning of his "Preliminary Observations," with which the work regularly opens, so that the reader would find at once the "Argument" or view of the range which the author intended to take, and the outlines which he proposed to fill up. The title and the preface tell this; but a distinct declaration, that the history of yellow fever, wherever it has displayed itself in different parts of the world, would be given, in connection with, and as an extended commentary on its forms of exhibition in Philadelphia, might well have preceded, as a general opening, the first topic discussed, viz: the Medical Topography of this city. But whatever, in the way of minor criticism, may be said as to the fashion of announcement of the course intended to be pursued, there cannot be much difference of opinion as to the propriety of the one actually taken. The historical sketch of the successive epidemics, in the shape of yellow fever, which have appeared in Philadelphia from 1699 to 1853, both inclusive, together with an account of the Medical Topography, Climate and Population of the city, constitute an appropriate introduction, and, as it were, a preparation for entering on the study of the disease in its world-wide visitations. The actual connection between the fever and certain localities, season, and temperature, and its reputed importation and contagion, are questions with which the reader, after a perusal of the "Preliminary Observations," becomes familiar; and for the full investigation of which he will be, in consequence, the better prepared to follow the author in different chapters of the body of the work.

This historical sketch possesses value in another point of view, viz., as a part of general history, and as a model for writers on both endemic and epidemic diseases, who ought to feel the necessity of combining a description of the place (region or city) and its fixed climatic character, with a narrative of these diseases and the associated and resulting circumstances of which it has been the theatre. Dr. La Roche in these respects follows worthily the advice and example set by our great master and teacher,

Hippocrates, who is so often referred to and so little studied and known by his reputed followers.

Being fully impressed with the importance of the matters treated of in the Preliminary Observations, and believing that our distant readers, and all indeed who have not visited Philadelphia, will take an interest in the topographical description and narrative of its epidemics of yellow fever, we shall introduce here the prominent portions relating to these subjects.

*Situation of Philadelphia.*—This city is situated on the river Delaware in 39°.37′ N. lat., and 75°.8 W. long., 120 miles, by the course of the river, from the Atlantic ocean, and sixty miles in a direct line to the mouth of the river ; or 48 in a line through New Jersey. It lies between the rivers Delaware and Schuylkill, "on a modern tertiary formation, consisting of sand and gravel, for the most part overlaid with a thick stratum of clay, of various hues and degrees of tenacity, the whole resting upon a primitive basis which lies at the distance of from forty to fifty feet beneath the surface, but shows itself in some of the northern districts." Various marine deposits show that the entire site of the city was once covered by the sea. Water is easily obtained in any part of the city, at depths varying from ten or twelve to thirty feet. The elevation of the soil above low water mark varies from two to forty-six feet, "the most elevated portion being about the third of the way between the Delaware and Schuylkill." The ground rises in the northwest and along the banks of the Schuylkill in that direction, and declines to the south to the point of junction of the two rivers. The tract between this latter and the city is called the Neck, and is low, flat and marshy : it is, for the most part, meadow land, and the remainder is laid out in gardens, from which the city is largely supplied with fruits, and still more with vegetables.

"The Delaware, opposite to Philadelphia, is about one mile in width, and of sufficient depth to admit vessels of heavy burden. It rises and falls with the tide between five and six feet in common times ; but, during a long continuance of violent north winds, it occasionally reaches a height of from seven to nine feet beyond its common level. It flows at the rate of from two and a half to five miles an hour ; the tide running seven hours up and five down."

The author next speaks of the ground plot of Philadelphia, the well graded and paved streets, and commodious side walks, the gutters and sewers communicating with the river. The houses of recent construction are supplied with water closets ; but more commonly the privies are in the yard or garden, their wells being dug sufficiently deep to reach a vein of water. The city is supplied with an abundance of good water from the Schuylkill.

Exceptions to the generally healthful arrangement of the streets and proper ventilation, are pointed out in the case of Water street, "which plays an important part in most of our yellow fever epidemics." It runs along and under the original bank of the Delaware, from one extremity of the city to the other. The houses on the western side of the street are built against the elevated ground, which was once the bank of the river, and hence the lower story at the back part is constantly damp, and even wet, at times, by the percolation of moisture from the earth, against which the lower part of the wall rests. There is, of course, no yard room, and the privies are placed in the cellar. On the other or eastern side of the street, nearest to the river, the houses back on the stores and warehouses, which are built along the wharves facing the river ; and here again is a want of common space for yard, so as to allow of ventilation, or the construction even of privies. The street itself is narrow and lined on both sides with high houses, once occupied by the better and some of the wealthier classes ; but for a long time past they have been either converted into warehouses and petty shops, or are tenanted by lately arrived emigrants, poverty stricken residents and sailors, &c. The whole of Water street is an encroachment on the original plan of Philadelphia, as laid down by Penn ; and scarcely ever has an infraction of the laws of hygiene, as it is, been so severely punished by the fatal diseases, especially yellow fever, of which it has been the frequent seat.

Other exceptions to the harmony of plan "as regards the general arrangement, convenience, and healthful construction of the city, consist in a number of alleys, closed courts, lanes, or narrow streets, by which the blocks of buildings (or squares, as we denominate the space extending from one street to another), originally laid out, are in some places subdivided. These receptacles, which are more frequently found in the southern and northwestern portions of the city proper, and still oftener in some parts of the suburbs, are occupied by the black and poorer classes of the population; by stables, and other establishments of the kind. Alleys of twenty feet wide are paved and lighted; the houses have yards and privies; but, in the smaller ones, nothing or little of this is to be found. They are not paved, and seldom cleansed; they are ill-ventilated, compactly built, in many places with small badly-constructed frame houses, many of which are in a state of dilapidation and crowded, and present often a melancholy picture of filthy wretchedness and misery."

The construction and distribution of the wharves along the Delaware, on the city front, are thus described:—

"The wharves, or quays, which now extend several miles along the margin of the Delaware, are built of square casements of logs, filled up with earth, vessel-ballast, and stones. The surface of these wharves, as also of the street running all along the river, is paved—an improvement of the last twenty years only—while the street itself, which, from the projection of many buildings, was both irregular and inconvenient, has been, by means of funds bequeathed for that purpose by the late Stephen Girard, straightened, in many places widened, and, in the main, greatly improved. The wharves so constructed project, in many places, into the river, leaving open spaces or docks between the projections, where vessels inclosed are protected from the destructive effects resulting from the action of the floating ice. But while some advantages accrue from the protection thus afforded, they are counter-balanced, whatever care may be taken, by inconveniences of a serious character in a hygienic point of view; for these recesses easily become receptacles of filth, and the wharves generally being elevated above the highest tides, it follows that, at low water-mark, their sides, and the half-dried surface of the docks, are exposed to the vertical rays of the sun, and become thereby the source of offensive, and at times injurious effluvia."

On the subject of interments in the city limits in former times and at suburban spots, of late years, we read as follows:—

"For a long while after the settlement of Philadelphia, the practice of city interments was exclusively followed; the grave yards used for that purpose being usually situated around their respective churches, sometimes in detached spots. In progress of time, the accumulation of dead remains has become considerable in many of the places of burial; in some of which it has been thought necessary to place stratum on stratum of earth, so as to make room for additional supplies of bodies. During the last twenty or thirty years, some cemeteries have been converted into open squares or promenades; others have been partially abandoned; while several establishments, of greater or less extent—some of them of considerable beauty—have been made at various distances around the city."

Six large open squares, five of which are laid out in walks and planted with forest and ornamental trees, and one of these embellished by a fountain, give space for ventilation and furnish promenades for health and recreation. The sixth, once known as *Centre Square*, and originally the largest of all, has been spoiled by being cut up into four divisions, which now go under the name of Penn Square.

The whole history of Philadelphia is comprised within a period of one hundred and seventy-three years, viz: between 1682 and 1855. This fact mentioned by the author, suggests our making the remark, that an estimable citizen and friend of his, is now in his ninety-sixth year, and consequently if at the birth of this gentleman his grandsire had attained the age of eighty years, the latter could not have been born on this side of the Atlantic. How rapidly can we run over the short list of the successive generations of natives, from the first born, after the landing of William Penn, down to the present time! The great grandson of one of the first settlers, whose name is given in the work before us, is now in the prime of life, thus making but the third step in the line of Pennsylvanian or American nativity.

The city plot was once partially traversed by small streams, the chief of which, Dock Creek, has its bed now occupied by Dock street and the culvert underneath. This street is one of the very few whose direction is not in a straight line. The creek ran from a spot now represented by that part of Third near Walnut street, and emptied into the Delaware at Front street, a little south of Spruce. The entire space represented by Dock street, and that extending from the latter to Spruce, was, in early times, a miry swamp, traversed by the sluggish Dock creek. This latter, after a while, with the increase of houses in the neighborhood, was made the receptacle of the refuse water and other matters from the several tanneries, and of the offals from the gutters and other sources, and, as a consequence, it became highly offensive by its congregated odors, and, still more, a source of disease. At an early period, the creek was arched over in the neighborhood of Third and Walnut streets; "and, in 1784, the arch was continued, principally through the active efforts of the late Doctor Rush, as far down as Spruce street, and the surface converted to its present use. For many years subsequently the arch and drawbridge [at the mouth of the creek] existed as heretofore, and were made use of. But, finally, the latter was removed, while the arch itself was filled up effectually, excepting always, an arched culvert under the pavement, through which the water of the creek and the contents of several sewers are discharged into the river."

Pools and ponds were met with in different directions; some of them of natural formation, others made by excavations for procuring clay for brick making.

"Besides these sources of morbific exhalations, there were others, both beyond the limits of the city and within its very precincts. A small stream of water, called *Pegg's Run*, passes through a portion of the Northern Liberties and Spring Garden, which, until a few years ago, was left open and unimproved. The bottom of the stream was miry, and, at low tide and in hot and droughty weather, was often destitute of sufficient water to carry off its contents. Receiving the offals of many slaughter-houses, tanyards, glue, starch, dressed skin, and soap manufactories adjoining it, as well as the contents of two culverts, of a large number of privies, and of the gutters of the numerous populous streets and alleys it crosses, it became highly offensive, and the source of noxious exhalations. This stream, which plays a conspicuous part in the history of one of the epidemics, and was correctly pronounced *the greatest nuisance in Philadelphia*, attracted finally the attention of the public and council, and has since been culverted.*

"Between the city and Kensington, the surface was long covered with stagnant water and dams; while, beyond the village, was banked land of a large extent.† In regard to these, some improvements have been made; but, in many parts, the bed is still covered at high tide, and remains muddy and marshy when the water recedes. So late as 1797, Dr. Pascalis writes: 'In the Northern Liberties and the district of Southwark, there are many vacancies on the banks of the river. Owing to the periodical floods, these form large miry grounds which are never dry, but covered either with thick beds of filth from the adjoining streets or habitations, or with rubbish, old timber, &c.' "‡

In addition to the sources of disease already enumerated, the author specifies the bad construction of privies. He then points out, as great improvements in the sanitary condition of the city, the general paving of the streets, and the abundant supply of good water. Little was done, however, until of late years, to prevent the wharves and adjoining streets from becoming frequent and prolific sources of disease. In reference to the improvements in these respects the author remarks: "All this has been the subject of observation among us, and no one has failed to perceive, that, by these various causes and the gradual spread of

* Jackson, Fever of 1820, p. 47, and Appendix.

† Barnwell, 368. ‡ Fever of 1797, pp. 94, 95.

improvement, malarial fevers have been cleared beyond the limits of the city, north, south and west, and the sources of noxious effluvia on the east [or Delaware] front greatly lessened."

But, although, much has been done, much yet remains undone. Morbid influences still exist, and while always more or less operative, they may, at a time when least expected, become fearfully so.

"They depend on the natural, uneven, and irregular condition of the city plot; on the vicinage of the river side; the peculiar quality, natural and artificial, of the soil, in many parts at least of its surface; the compact construction of the houses; and the narrowness, imperfect ventilation, and filthiness of the streets in certain sections of the city. They arise also from the numerous courts and alleys, many of which are closed at one end, unprovided with a sufficiency of water, imperfectly drained, often unpaved, to say nothing of the piggeries and other nuisances they contain; the denseness and squalid character of the population of these localities; the defective mode of construction, and the physical peculiarities of wharves, built of perishable timber, and filled up with animal and vegetable remains and rubbish of various kinds; as well as from the condition of the docks, and the proximity of the shipping. Objectionable localities—though more numerous, perhaps, formerly than they are at present—may be found in almost every district, even in the very heart of the city, but abound more in some portions of it than others."

The *climate* of Philadelphia, is truly described by the author to be, like that of the surrounding country, one of extremes: "the cold of winter as well as the heat of summer, being almost invariably considerable, and at times, of very great intensity." After speaking of the general character of each month of the year and of the seasons in succession, he arrives at the following conclusions: "That, notwithstanding the extreme heat we suffer during the summer, coldness is the predominant feature of our climate, as there are not, in all the year, more than four months—one third of the time—in which the weather is agreeable without a fire."

"As regards the range of atmospheric heat observed among us, we may safely affirm, that it rarely happens that more than twenty or thirty days present themselves in summer or winter in which the mercury in Farenheit's thermometer attains a height of more than 80° in the former, or falls below 30° in the latter season.* From a review of the results of observations made during a long succession of years—from 1758 to 1848 inclusive—it will be found that the annual averages varied from 48° (1836) to 56° (1819);† giving a mean temperature throughout the

* Rush, 81.

† Summary of College of Phys., i. 340, ii. 361; Hewson, Am. Phil. Tr., vi. 395–8; Forry, Climate of United States, p. 10.

year, for the whole series, of 53°—this being the temperature of the deepest well-water."

The extremes of temperature show themselves, by the thermometer; rising in the greatest heats of summer, during a succession of days to 90° and 96° F., and sometimes to 100° F.; "while in the winter months, it descends often as low as zero, and even, at times, several degrees (varying from 3 to 7) below this point." The more usual elevation of the mercury, however, in the hottest period of summer is to 85° or 86° F., and the greatest descent in the coldest weather is seldom less than 5° below zero. The difference between the extremes of temperature in the summer and winter months, may be rated at 80° F.

"It will be found, on examining the whole number of observations from 1758 to 1854, that of the twelve months, the one which, by its mean amount of heat, expresses the nearest equivalent to the mean annual temperature, is October; a result corresponding to that pointed out by Humboldt in reference to modified climates generally. The months which exceed the annual mean are May, June, July, August and September; while those that fall below are January, February, March, April, November, and December."

The temperature of Philadelphia is little inferior in mean results, and superior as to extremes to that of the coast of South America, the West Indies and even the coast of Africa. "In a word, while our winters are almost Siberian in character, our summers are, as already stated, truly tropical, and capable, therefore, when aided by the conditions of locality already mentioned, and by other morbific agencies presently to be adverted to, of giving rise to disorders peculiar to hot climates."

"The barometer exhibits a mean elevation of about 30°; the variations being very inconsiderable in the greatest changes of weather."

The average yearly quantity of rain during a period of 44 years, was 38 inches. According to a series of observations on this point, by Dr. John Conrad, made during a period of 25 years, the annual average was 43·76: the spring and summer representing the months of April, May, June, July and August are often the wettest in the year. From the records of the rain guage, kept at the Health Office, in a central part of the city, from March 1820 to February 1827 inclusive, the three months of winter averaged 8·15; spring, 8·29; summer, 9·54; and autumn, 10·54.

As regards serenity of sky, Philadelphia will compare advantageously with cities which we are accustomed to speak of, as remarkable in this respect. "Thus, while clear or partially clear days constitute more than two-thirds of the whole number throughout the year, at Naples they do not exceed the one-fifth or one-fourth of the whole. The mean number of rainy days at Rome and Florence, is 114; at Sienna, 104."

The dew point in Philadelphia, averaged, during five years, or from 1844 to 1848, both inclusive, 41°.51, the variations in different years were from 29° to 62°, 25° to 62°, 21° to 65°, 24° to 64°, 25° to 67°, the dew point being much higher in the warm than in the cold months. "It usually commences to rise about March and April; continues to rise until it attains its maximum in July and August, and then again descends."

The author next speaks of the population of Philadelphia, and its progressive increase from 1683, when it was only 504. At the beginning of the next century (1700), it was 4,417, and in 150 years after, or in 1850 it was 408,862, including the neighboring towns and villages in the county. A table is given of the progressive increase of population in many of the intermediate years. The proportion of the inhabitants of African descent to that of the whites in 1850 was 1 to 20·69. In the city proper the actual numbers of the colored race was 10·736 or 1 in 10·36, while in the county they numbered 19·761.

After claiming for Philadelphia, on good grounds, the character of healthfulness, compared with other cities of equal size, the author points out the yet existing and common causes of diseases, and contrasts their more potential operation in former times with their diminished force in our own day. Remittent and intermittent fevers once common in nearly all parts of the city are now limited to the suburbs. "In former days, when the city was of limited extent—with few improvements—with buildings scattered about, and leaving open and unimproved spaces between—with a marshy stream running through the greatest part of it—with ponds, natural and artificial, spotted over the plot in various directions, and with unpaved streets—fever was of common occurrence, and epidemics not unfrequent. At present, malarial fevers are unknown in the city proper, as well as in the compactly built and well drained portions of the suburban districts."

After a detailed account of the Medical Topography of Philadelphia, a connected abstract of which we have endeavored to place before our readers, the author gives a historical sketch of the successive epidemics of yellow fever, as they appeared in the years 1699, 1741, 1747, 1762, 1793, 1794, 1797, 1798, 1799, 1802, 1803, 1805, 1820 and 1853. Gladly would we follow him in this retrospect, did our space permit. The narrative of the earlier attacks of the pestilence, necessarily brief from the scantiness of materials, acquires more fulness, as these increase, down to our own time. It is, throughout, plain and unpretending, without the declamation, rhetorical flourishes, or attempts at pathetic description, in which a less careful and less philosphical writercould hardly refrain from, at least occasionally, indulging. It is, notwithstanding, replete with interest, even to the general reader, both by the importance of the theme and the natural account of the scenes to which the disease gave rise, as described by contemporary writers and observers.

The first epidemic, in 1699, was attended with a mortality fully equal to or exceeding in its proportions any that has occurred in subsequent times. The population at this date, must have been considerably less than 4,000 inhabitants. Story, one of the few who have left any description of the fever, "states that the mortality amounted to six, seven and eight a day, for several weeks, and that ten were buried in one day. The entire lack of medical testimony respecting the disease, is, in part, supplied by the letters of Isaac Norris, great-grandfather of Dr. W. Norris, Wm. Monington, and extracts from the Journal of Thomas Story, quoted above. Mr. Norris describes the fever as the Barbadoes distemper. The symptoms described by this gentleman and by Mr. Monington leave the inference in the mind of Dr. La Roche, that the fever differed in nothing from that which, under the name of yellow fever, has subsequently prevailed in Philadelphia. "The epidemic of 1699 was preceded by one of influenza.* Like those that have occurred subsequently, it limited its ravages almost exclusively within the precincts of the infant city, and

* Webster, i. p. 212.

extended but a little, if at all, into the country."* No where does the author find a suggestion that any vessels had arrived from the West Indies with the disease on board, or in a condition calculated to infect the city. At a later day, Gough and Proud threw out the idea that it was the offspring of foreign importation; and Pemberton, appealing to the reminiscences of his father, who was then but fifteen years of age, asserts, "that it was imported in a ship (or other veesel) from the Island of Barbadoes, whose cargo consisted of cotton in bags, which were landed at a wharf between Market street and the drawbridge, and there stored for sale."

In reference to the appearance of the yellow fever in other parts of the world, during this century, the author tells us, on authorities cited, that it existed in Barbadoes in 1647, in St. Christopher in 1648, and in Jamaica in 1671, at Leogane (St. Domingo) in 1691, in Pernambuco (Brazil) from 1687 to 1694, in Martinique, in 1690, in the city of the Cape St. Domingo in 1696, and at Leogane in 1698. Barbadoes was afflicted by it in 1691, 1694 and 1696. "An epidemic of fever, upon the true yellow fever nature of which there can be no doubt, prevailed at Rocheford, in France, during the autumn of the same year, 1694."

"In the same year that the yellow fever made its appearance for the first time, in Philadelphia, it prevailed to a considerable extent in Charleston, where, as we are told by Hewatt,† it carried off a considerable number of people."

A long interval of exemption from yellow fever, extending through a period of forty-two years, or from 1699 to 1741, was enjoyed by Phildelaphia. "The records of the period show that it prevailed, with more or less severity, at Martinique in 1703 and 1706, at Cape François, now Cape Haytien, in 1705, 1723, 1733, 1734, 1739, and 1740, at Leogane in 1732; at Carthagena in 1729 and 1730, at Barbadoes in 1723 and 1733. At Charleston it prevailed in 1703, at which time the inhabitants were apprehensive of an invasion of the French and Spaniards. It broke out again with great violence in 1728." "It again appeared in 1733, 1739 and 1740."

* Story, 223.

† An Historical Account of the Rise and Progress of the Colonies of South Carolina and Georgia. i. p. 142.

In Philadelphia, the fever of 1741 broke out with violence, and continued until it was ended in its progress by the coming on of cold weather. The disease prevailed in Jamaica and other West India islands, during this year. The information, as we learn from the author, respecting the epidemic of 1741 is still more scanty than that of 1699. He has gleaned, however, many interesting particulars, for which we refer to the work itself, tending to show its identity with the fever prevailing, at the same time, in the West Indies, New York and Virginia.

The disease in Philadelphia was generally attributed to foreign origin, but there was then, as has often occurred since, in the history of epidemic visitations of yellow fever, here and elsewhere, no little discrepancy of belief as to the place of origin and the manner of its introduction.

The occurrence of yellow fever in 1744 in Philadelphia when it prevailed to a great extent in New York, is doubted by the author. It is not so, however, with the outbreak of the disease in 1747, of which we have notices by different persons named, and among them Dr. Franklin. "As on other occasions, the fever broke out during the hottest part of summer. The disease was confined within the limits of the southern part of the city, below the drawbridge, and at lodgings for sailors and in the neighborhood of the dock which was then uncoverod." A belief in the contagiousness of the fever, and of its having been imported, was very general.

Philadelphia was slightly visited by the yellow fever in 1760, and in a more decided manner, and with more fatal results in 1762. "The disease made its appearance at the usual period of the year; it was preceded, as all epidemics of the kind usually are, by hot weather, was circumscribed within such localities as it has been ever found to prefer, and was arrested by the accession of winter." A specification of its actual range and limits is given in a subsequent passage by the author. All that we possess of clinical observations on this fever, is found in the notebook of Dr. Rush, and a short sketch communicated to the College of Physicians, by Dr. Redman. As in former instances, we find this fever attributed to a foreign source. "The time had not yet come, when it could be admitted to originate from the baneful action of foul local exhalations. But as usual, opinion differed as to the mode of its introduction, and the place whence it came."

After a long interval of thirty-one years, the yellow fever broke out in Philadelphia in 1793, with an intensity and a resulting mortality greater than at any former or (except 1798) subsequent year. Its history, unlike that of preceding epidemics in this city, is not required to be made out by circumstantial evidence, but comes from numerous, well written and detailed descriptions, by those who saw what they described, and who bore a more or less conspicuous part, in their professional and philanthropic efforts, for the mitigation and cure of the disease. Dr. La Roche has turned his materials to good account in his succinct yet clear narrative of the leading incidents of this memorable pestilence. He describes, after Dr. Rush, the state of the weather during the preceding part of the year, down to the date of the appearance of the disease about the middle of August. The earlier notices of its attacks were treated by the people, generally, with incredulity mixed with anger. The disease made its appearance about the middle of August. Its geographical range is given, and the remark made at the same time, "that during the whole course of the epidemic, the greater number of the cases occurred in the vicinity of the Delaware river; that there, also, as in close alleys and small streets, the disease assumed its most aggravated form, and proved most unusually fatal; and that its severity lessened in its progress westwards and towards the districts."

We do not pretend, either by extracts or condensed narrative, to convey to our readers a connected view of the progress of the fever, and the concomitant gloom, despondency and despair, with instances of heartless selfishness, redeemed in others by traits of rare devotion and philanthropy—as described in the volume before us. That which, by the pen of the author, has been given with brevity and condensation cannot well be abridged by his reviewer; and hence, we must refer the reader to the fifteen full pages of the volume itself, for the desired information on this eventful theme. We would, at the same time, extend the reference beyond the medical pale to intelligent readers of history, sure as we are, that in the incidents of the present one, thus simply and plainly told they will find abundant material both to interest their feelings and to add to their stock of general knowledge. The mortality from the yellow fever epidemic of 1793 has been variously estimated as from 1 in 10 to 1 in 12·13 of the whole population, taking the two estimates of the latter at 40,000 and 50,000. The entire number of deaths was a little over 4,000, of which, the largest number in the four months of its continuance was in October, being 1976. In reference to the proportionate mortality of those who were sufferers from the disease, and to the entire number who were attacked, the author remarks: "At a future time, this subject will be resumed, when reasons will be given for believing that the proportion was about 1 to 3, and that the number attacked amounted to near 11,000."

The mortality great as it appears by the above calculation is rendered still greater, when we bear in mind, that, at the time of the enumeration 2,728 houses were closed, and not, less than 12,297 of the inhabitants, (Cary says 17,000,) had fled the city and sought refuge in the purer air of the country, or been removed there by the public authorities.

When it is known that, with few exceptions, the medical men remained at their posts, in the active and zealous performance of their duties, we can well understand how it was, that amid continued exposures and the interruptions in their hours of sleep and meals, and the slightly remitting strain of mind and body, so many of them should have fallen victims to the pestilence. "In the space of five or six weeks, exclusive of medical students, not less than ten physicians were swept away by the epidemic. Scarcely one of those that survived or remained in the city escaped sickness." At one time, if we except the French practitioners, then residing in the city, there were but three physicians who were able to do duty out of their houses.

The year 1793 was a memorable one in the medical history of our yellow fever, as that in which, for the first time, men of mark and authority avowed their dissent from the belief of its being an imported disease. Among these gentlemen we find the names of Drs. Rush, Redman, Foulke, Leib and Hutchinson. The doctrine of contagion, previously received, was disputed at this time. "From this moment may be dated the origin of the interminable dispute about contagion and non-contagion, which has continued ever since to occupy the attention of the medical profession in this and other countries."

Dr. La Roche does not enter thus early into an examination of this mooted point; but the importance which he attaches to its solution is evinced in the fact of his devoting nearly four hundred pages to it in the second volume of the present work.

Next comes a brief notice of the yellow fever of 1794, and a more particular one of that of 1797, which latter was attended with considerable mortality. "The condition of the localities where the disease appeared and was mostly prevalent, differed in nothing from what it was on former visitations. The Academy of Medicine, in a letter to the governor, called attention to the putrid exhalations from the gutters, streets, ponds and marshy grounds in the neighborhood of the city, and traced to these sources most of the cases which occurred in the city and at Kensington Bridge.

As on a former occasion, medical men suffered largely from this fever. Out of 23 or 24, the entire number in attendance on the sick with yellow fever, nine fell victims to the disease.

The fever of 1797 was, as before, attributed to importation. This opinion was formally advanced by the College of Physicians, and the origin of the fever was traced, according to this authority, to two vessels, the one from Havana, the other from Port au Prince. On the other hand, thirteen physicians, "in two letters to the Governor of the State, the one in their private capacity, the other as members of the 'Academy of Medicine,' discarded the idea of importation, and advocated decidedly the local origin of the disease, referring it to putrid exhalations, and to the fact that two ships that had arrived from Marseilles and Hamburgh, had, while discharging their cargoes, infected the vicinity of the wharves, the former at the foot of Pine street and the other at Kensington. They enumerated all the common sources of malignant fevers, and recommended removal from the city as the most effectual method of guarding against a renewal of the disease."

Still more formidable by its malignity and the mortality to which it gave rise, was the yellow fever of 1798. In these respects it went beyond any preceding epidemic visitation. The mean temperature of the three summer months—June, July and

August—was, as we learn from the volume before us, not lower at mid-day than 79° 94′ F., an elevation greater by several degrees than that observed in other yellow fever seasons, but below that of 1793, when it rose to 81° 37′ F. During the season the thermometer rose forty-one times after mid-day (3 P. M.) to above 80° F.

"The localities where the fever made its appearance and prevailed, were in no better condition, in point of cleanliness, than those that had been the theatre of its ravages at former periods. The sinks and sewers, were as heretofore, filled with putrefying materials and emitted the same effluvia." Here follows an enumeration of the chief of these nuisances.

The disease began this year early in August—"Assuming rapidly a fearful character of malignity, and spreading far and wide, it prevailed from that period through the rest of the summer and during the whole of autumn." The month of July had been marked by cholera morbus, especially among children, and by dysentery and several cases of bilious fever of a highly malignant grade.

Resembling in its range former epidemics, the fever was principally met with along the river side and in the adjoining streets. It began about Spruce and Walnut streets, and the wharves; but after a short time, and before the close of August it spread nearly throughout the part of the then city, as far as the jail, situated at the corner of Sixth and Walnut streets, and even into the Pennsylvania Hospital, in Pine, between Eighth and Ninth Streets.

The proportionate mortality among those attacked by the disease, and who continued to be exposed to the infection, was greater than in 1793. The population of Philadelphia was estimated in 1798, to be 60,000, but such was the alarm excited by the appearance of the fever, and such the urgent advice of the College of Physicians, the Board of Health and the Academy of Medicine, that according to an accredited computation, three-fourths to five-sixths of the inhabitants fled the city to the neighboring small towns and adjoining country. By this means a comparatively small number were exposed to the infection and to share the loss occasioned by it. Thus while, as we learn from the author, the deaths in proportion to the entire population were

1 to 16.48, they were, in fact, in a much greater proportion to the actual and vastly diminished population caused by the emigration just noticed; as but 20,000 inhabitants were left to bear the brunt of the attack of the pestilence, and their loss by deaths would be one out of every five or six persons. The mortality by death among those attacked by the disease was excessive. The latter numbered 4.71, including 898 received from the City Hospital: the former 3,645, making a proportion of deaths to cases as 1 to about 1.27.

The explanation of this fearful mortality is found by the author in the great diffusion of the poison, and also in the condition of the inhabitants, chiefly the poorer classes, who were left in the city, and who resided in confined, ill-ventilated and filthy courts and alleys, and of course exposed in an unusual manner to the influence of morbific causes. The sufferings of the poor and destitute on that occasion, aggravated as they were by the dread of contagion and the general panic, are briefly but well described in the work before us.

The following quotation furnishes matter for melancholy reflection, when we compare the positive experience and enlarged humanity of the people in the towns and rural districts near and around Philadelphia in 1798, with the theoretical notions and their cruel fruits by which the inhabitants of Norfolk and Portsmouth, fleeing from the ravages of pestilence, were driven back and refused shelter and even a resting place by the people of their own State, in this present year of our Lord, 1855.

Dr. La Roche says, "Experience had already shown that no fear of contagious communication was to be apprehended from the effect of emigration." From this period may be dated the revolution in Dr. Rush's mind relative to contagion, which he no longer believed, and which he took occasion a few years afterwards frankly to avow.

The fever of 1799 penetrated almost every part of the city east of Seventh Street, beyond which very few cases occurred; it chiefly "affected the wharves between Pine and Lombard Streets near the southern boundaries of the city, and the District of Southwark, in the vicinity of the Swedes Church, (*Currie*, p. 5.) In this season, the village of Kensington, which, in former epidemics had usually suffered, was spared." Notwithstanding the wide spread of the disease, the deaths barely exceeded a thousand.

The yellow fever showed itself only in a sporadic form in Philadelphia in the years 1800 and 1801. Other cities of the Union were less fortunate. "In 1800, the fever prevailed extensively in New York, Providence, R. I., Norfolk, Baltimore and Charleston; and every medical reader must recollect the devastation produced by it in Cadiz, Seville and other cities of Spain; and in 1801, it broke out in several of the cities of New England, and spread so generally in New York, as to produce a considerable desertion of the city."

The yellow fever was epidemic in 1802 and 1803 in Philadelphia. In the first of these years, it broke out on the 15th of July, "near the corner of Front and Vine streets. On the 2d of August, it extended to other parts of the city, particularly Front and Water streets, near the Drawbridge, as well as to Chestnut street, near the wharf." "The fever declined after a black frost which occurred on the 23d of October, and ceased entirely in November."

The principal field of the fever of 1803 extended from Market to Walnut streets, and from the east side of Front to the Delaware. There the disease assumed a most malignant character. It became milder as it exhibited itself westwardly. "The whole of the city and liberties, except that portion which lies between Second street and the Delaware, remained as free from disease as it has ever been known in the most healthy seasons."

This is a noticeable year in the literature of our subject, as one "in which the doctrine of local origin and non-contagion appears to have made some progress." The Board of Health "in issuing a declaration of the existence of the disease, affirmed that it was not contagious, and this opinion was entertained by Drs. Rush and Caldwell, and other leading physicians. By others, however, and the public at large, the old and contrary doctrine was maintained with as much pertinacity as ever."

The appearance of the yellow fever in 1805 in this city, was preceded by its breaking out in 1804, "at Leghorn, and in not less than twenty-five cities or towns of Spain—Cadiz, Ecija, Carthagena, Malaga, Alicant, Gibraltar, &c." It prevailed, also, at this time to a considerable extent in Charleston and New

York. In Philadelphia, its attack in 1805 was contemporaneous with that in New York, New Haven and Providence. The author draws freely in his account of the epidemic from the well written history of the disease by Dr. Caldwell.

In the notice, by the author, of the fever of 1820, he makes free use of the elaborate papers on the subject, written by Professor Samuel Jackson, which are the result of the formal observations at the time and the subsequent readings of the latter. The whole scope of the experience and study of both the gentlemen just named, is adverse to a belief in the importation or foreign origin, and in the contagion of yellow fever.

A long period of exemption from this disease was enjoyed after 1820; and it was not until 1853, or after an interval of thirty-three years that Philadelphia was somewhat frightened from its propriety by some unpleasant demonstrations on the part of its old enemy. The number attacked was only 170, of whom 129 died, making the mortality 79 per cent. There is a good reason for believing, with Dr. Jewell, that not all the cases, particularly those of a milder character, were reported. The period from the date of the first announcement of cases, July 19th to October 7th, when the last case was reported, was two months and nineteen days.

After the statements made by Dr. Jewell, of the home sources of febrile disease, in the outlet of the sewer into the dock at South street ferry, "belching forth continually putrid masses of animal and vegetable filth," and a most foul wharf at the upper side of South street, "numerous damp and confined cellars, subject to an occasional overflow by the ebbing and flowing of the tide-water of the Delaware," and other minor causes of the same character; added to the state of the gutters, alleys, &c., in other places where the disease appears to have originated, it is difficult to give credit to the assumption of foreign origin, in its being imported in the barque Mandarin, from Cienfuegos, Cuba, which discharged her cargo at a spot in the neighborhood at which the fever first appeared. No adequate evidence was furnished of the disease exhibiting a contagious character. Dr. Jewell gives no countenance to such a doctrine, although he believes in the exotic origin of the fever, in the form of a poison generated in the imber planks of the hold of the Mandarin, which was afterwards diffused through the air at the wharf and neighborhood, and acquired increased strength by meeting with other elements of decomposition; the whole acted on by high heat and moisture.

The picture of Philadelphia in its climate, situation and dark spots, and the endemic visitations of yellow fever, so ably depicted by the author of the present work in the Preliminary Observations, and of which, on account of its importance and subsequent applications, we have given an outline sketch, will represent in the main, and, indeed, in essential particulars, the medical topography of other places in which the disease has prevailed. These are, high temperature of the summer months, often continued heat, approximating the climate to a tropical one, defective sewerage, and accumulations of vegetable and animal matter acted on, and, in a degree, volatilized by heat and moisture, narrow and filthy streets, confined, damp and badly ventilated, and sometimes underground habitations. These are features common to nearly all, if not all, the places which have at different times been ravaged by the disease. To repeat a description of them was unnecessary, and would have swelled the work to unreasonable dimensions. Accordingly, the author proceeds to a methodical and descriptive account of the several elements or parts which make up yellow fever; beginning with a general description of the several species, and continuing with a particular one of the symptoms, separately examined, through the range of inflammatory and congestive species, with their several subdivisions, and also the premonitions and modes of invasion. Next, we have the symptoms deduced from the circulating system, by the pulse, and hemorrhages, external and internal; from the state of the skin, as measured by its temperature, secretions, odor, sensibility and color, (including jaundice,) eruptions of various kinds, abscesses, anthrax and gangrene; from the digestive apparatus, as indicated by nausea and vomiting, and the alvine evacuations, and the fearful black vomit, also, by the tongue and thirst; from the respiration, and from pain; from the countenance; from the urine; from restlessness and jactitation; and wakefulness and muscular power. The symptomatology is concluded by an analysis of the value of the symptoms furnished by the nervous system, viz., delirium, convulsions and spasms, carpologia, subsultus tendinum and tetanic symptoms. Then follows a view of the pathological anatomy of yellow fever, and of the connection between its anatomical characters and the symptoms. Critical days and critical efforts, and the type and complications of yellow fever receive, severally, a chapter. Duration, convalescence and relapse make up another. The several subjects just enumerated constitute so many themes for description and comparative investigations, which are conducted with the care, attention to accuracy and detail observable in all parts of the work. They occupy twenty chapters and 350 pages of the first volume.

The two chapters which open the volume are, the first on the synonymes, and the altitudinal and geographical ranges, and the second, on the classification of yellow fever. The range of the yellow fever is circumscribed within certain heights above the level of the ocean, and degrees of latitude north and south of the equator. Major Tulloch, quoted by the author, asserts that the disease has never been known in any climate to extend beyond the height of 2500 feet. Comparative exemption is enjoyed at less elevation in different spots in the West Indies. In Mexico, on the other hand, as Humboldt assures us, the farm of the Encero, at a height of 3,243 feet, forms the upper limit of the range of yellow fever, (the *vomito;*) and this disease scarcely ever passes beyond the ridge of mountains that separate La Guayra from the valley of the Caraccas. "In this country, [the United States,] the yellow fever is never known to prevail in very high situations, whatever may be the condition of the localities. But at what point it ceases to appear or prevail is still an unsettled question." To the explanation of the cause of the limitation of the range of the disease, we assent, with the exception of the concluding lines, viz., that "the liability of the yellow fever to be generated at a high elevation above the level of the sea, depends in part on the greater elasticity and purity of the air, on a diminution of atmospheric pressure, and on a more thorough ventilation. But the main cause is the absence there of the atmospheric heat, which, as we have seen, is indispensably necessary for the elaboration of the morbific agent to which the disease is due. For the same reason, in part, though not exclusively, its geographical limits are restricted to within certain limits, in a northern direction; while in a southern, the same effects are produced, as it would seem by an excess of heat and a variety of influences of a meteorological and telluric nature." The latter part of this sentence is obscure and contradictory. Taking the equinoctial as the dividing line, yellow fever is met with both to the north and to the south of this line, but it ceases to prevail at high latitudes in either direction, as we leave the equator and approach the poles, and for the same reason, viz., diminution of climatic or atmospheric temperature. To assume high heat as a cause of the limitation of range in a southern direction, by which the author means southern to us and nearer the equator, is in direct contradiction with the opinion incidentally expressed just before, viz., that high atmospheric heat is "indispensably necessary for the elaboration of the morbific agent to which the disease is due," and more formally enunciated at the conclusion of the chapter, when he says, "The yellow fever being a disease of hot weather, requiring a high average temperature during the summer months, and manifesting itself in no climate where the temperature is below that average, it is governed, in a great measure, in regard to its outbreak, duration and termination, by that condition of atmosphere." But, again, what are the limits, in a southern direction, of the fever? It is met with, as the author shows, below the 9th degree of north latitude, or at 8° 56′ on the Pacific.

Speaking of the identity of the yellow fever of Philadelphia, of the symptoms of which he had just before given a minute description, with the disease under the same name which has appeared and been described in other parts of this continent, the West Indies, and in Europe, the author writes, "In all places we discover that the disease is one of a single paroxysm. In all, we notice the characteristic appearance of the eye and countenance; the lull or stadium without fever of the second or third day. In all, we find the peculiar absence of febrile excitement after the subsidence of that stadium; the same gradual slowness of the pulse and depression of cutaneous heat in the after stage; the same coloration of the surface, the same character of the matter ejected from the stomach." An adverse opinion advanced by Dr. Richoux is refuted by the author.

On the subject of premonition, differences are pointed out in the fact that in some instances the fever is announced by hours and days of indisposition; in others, it comes on suddenly, and with little, if any, warning. Often, if not generally, the disease attacks in the midst of ordinary health, or is not preceded by perceptible indisposition. A somewhat qualifying view of the matter is given by Dr. Wragg, who had charge of the Roper's Hospital in Charleston, during 1854. From this, it appears that in 225 cases the attacks were sudden; in 92 they came on insidiously; in 32 the patients complained of malaise, &c., for a considerable time, and then gradually became feverish; and in 101 cases there were offered the usual symptoms characterizing the approach of fever. The difference in the premonitory symptoms depends, we are assured, in a great measure, on the mildness or violence of the attack; they being most evident in the former case, and nearly wanting in the latter.

The invasion of yellow fever was, in a large proportion of cases, marked by a chill; but, in this respect, there was no little difference in different years. Of 232 cases observed with reference to this particular point, "104 commenced with it, and 128 without." It may be added, "in some cases the disease is ushered in by giddiness, bearing great analogy to that produced by inebriating liquors." In other instances, it has been known to be ushered in with convulsions, syncope, &c. The attack usually comes on at night or towards morning, a period the coolest of the twenty-four hours, and one in which, according to the observations of Dr. Davy, the temperature of the body is lowest and the pulse commonly slower.

Among the symptoms furnished by the circulatory system, the changes in the blood are carefully investigated by the author; but without any very definite result, in the way of improving the pathology of the disease, or of confirming the somewhat popular idea, that to which the author evidently inclines, of the poison of yellow fever acting on and deteriorating the blood, and through this fluid the tissues and organs generally. The peculiar smell emitted from the serum of the blood drawn from yellow fever patients, and the elevated temperature of this latter fluid, which is some degrees above the common standard, are facts worthy of commemoration. The entire chapter on the morbid appearances of the blood in yellow fever should be carefully read, for its multiplied facts and the suggestions to which they give rise, even although, as yet, they fail to lead us to positively definite and satisfactory results.

The pulse in yellow fever is a fruitful theme, and it has received in the work before us a large share of attention. In the first stage of the disease, or in the beginning of the attack, the pulse is often strong, tense and full, and even bounding. In some instances it is "soft or feeble, though more or less full, or weak, depressed and small, and, at times, natural." The pulse is also very frequent, ranging from 80 to 100 in a minute; and again, it has been found to be slow, sluggish or natural: an intermitting or hobbling pulse is a not unfrequent symptom. The general conclusion, however, is that yellow fever is not a disease in which the arterial system is necessarily implicated, and hence, that the pulse, either as an aid to prognosis or a guide to practice, is not reliable. Dr. Pennell, in the epidemic of Rio Janeiro, in 1850, noted, with some care, the action of the heart—its impulse and sounds.

Among the symptoms of yellow fever furnished by the circulatory system, are hemorrhages. "Though not peculiar to that form of febrile diseases, they constitute one of its main characters. They take place in many mild or moderate cases; are absent in but few of the violent, malignant and fatal ones; and issue from a greater variety of parts than is found in most other complaints." The author, under the heads of external and internal, describes each of them with considerable minuteness, after a few remarks on their general character, and their influence on the march and termination of the disease. Among the external hemorrhages, that from the stomach, true hæmatemesis, as distinguished from black vomit, merits notice.

The second of the three chapters given to a consideration of the symptomatology of the skin, treats very fully of jaundice in connection with yellow fever. The odor of the skin, a symptom of some moment, though it has been frequently overlooked, is attributed by the author to that of the blood, which has undergone the poisoning process to a certain extent.

Jaundice, so common in yellow fever as to cause the disease to be designated by this color, is not by any means a constant attendant, since, in a large number of instances, the fever marches to its termination, either in health or death, without this symptom having been manifested at all. The entire subject, in its various bearings, is discussed with great fulness; and the strong probability that the peculiar changes in the blood and the subsequent effusion of the serum colored by the colliquation or dissolution of the red globules, are the cause of the jaundice, is maintained on various enumerated and plausible grounds.

Of the symptoms of yellow fever furnished by the digestive organs, black vomit is the most distinctive. It is, in fact, looked upon by many writers, as the great pathognomonic feature of the disease, the presence of which is essentially necessary to enable us to say that we have to deal with yellow fever. This extreme view is properly contested by the author, who shows that coffee ground ejections from the stomach, resembling, and of the same nature as the black vomit, are met with in other fevers and diseases, and that they are also caused by various poisons and the introduction of putrid substances into the circulation. Numerous interesting cases bearing on this point are related in this chapter of the present work. It is equally clear, on the other hand, that in a great many—even of the fatal cases of yellow fever, black vomit does not show itself. It has, indeed, great and melancholy significance; and in a semeiological point of view, its occurrence too generally preludes a fatal termination of the disease.

Worthy of remark, however, is the fact, that, although there may not have been any ejections of the matter called black vomit during life, it is found, in a great majority of cases, in the stomach after death. Dr. Blair, as quoted by the author, remarks, "that black vomit in the stomach is the rule, its absence the exception."

"The black vomit usually makes its appearance at the opening, or about the middle of the second stage of the disease, sometimes at the decline of the first stage; occasionally but rarely during the first or febrile paroxysm." "The most usual period of its appearance is about the fourth or fifth day. In some instances, it occurs earlier, and sometimes later." In the first contingency, on the second day; in the latter, as late as the seventh, eighth, or ninth day, or even much beyond.

Although so often a fatal sign, black vomit is sometimes followed by recovery from the fever. Numerous cases of this nature are related in the work before us. On occasions, the immediate effects of the discharge is a great relief to the patient; but for the most part this is illusory and of short duration.

Contrary to what happens in the first stage of the disease, in which retching and vomiting are associated with straining and much effort, the discharge of black vomit is made as if it were spouted out by the action of the stomach alone. The quantity discharged and found in this organ after death, varies of course, in different cases. Dr. Cathrall has known one gallon to be vomited in 24 hours, and Dr. Firth, among other observers quoted, "states, that the fluid is thrown up by pints, quarts and even gallons. In a case mentioned by Dr. Townsend, of New York, the quantity found in the stomach amounted to two quarts. The disparity between the quantity of the black vomit thrown up and that of the fluids swallowed is often noticed by the patients themselves."

The term black is not strictly correct, to designate the matter thrown up from the stomach. "As seen in this, city it is more frequently of a dark brown, bister, chocolate or amber hue. In some instances, the color approaches to a dark slate, or to a muddy claret color. It is of two kinds. The one consists of a number of dark flaky particles, which have been not unaptly compared to butterfly or bees wings, and which assume gradually the appearance, with more or less distinctness, of the grounds of coffee, of soot, or finely powdered charcoal, floating in a quantity more or less considerable, of thin glairy fluid, bearing a slight resemblance to a weak infusion of flaxseed, or green tea. The latter fluid, when filtered, differs slightly in color, being limpid like water, of a deep brandy or rum color, yellowish or light green." "The fluid, when completely formed, though homogeneous in appearance when discharged, separates soon, on standing, into two parts; the one consisting of the flaky or coffee ground matter, already mentioned, and the other of the fluid in which they were held in suspension."

"The other form of the black vomit, is more homogeneous in character, and presents the appearance of dark-colored inspissated mucus, or thin tar, or of a thick mixture of molasses and water. In some instances, the matter vomited, consists of grumous or dark colored blood, fluid or coagulated, without admixture of coffee ground particles, or pale fluid; while in others again, the matter described above, is mixed with coagula of more or less pure blood. In some instances, the discharge towards the termination of the disease becomes nearly sanguineous." The dark matter discharged from the bowels, is identical with that of the black vomit; and in fatal cases, it has been found, on dissection, covering portions of the inner surface of the intestines, especially the smaller ones.

Black vomit, usually spoken of as destitute of odor, has, in fact, a manifest character in this respect. The author, after citing the comparisons made by different writers, coincides from his own observations, with the statement of Dr. Hiester: "that it has a fresh, disagreeable, nauseous smell." This fluid, in general, is without the acridity attributed to it. In taste, the black vomit is more frequently acid, and such it is shown to be by reactives.

The question, yet a mooted one, as to the nature and real characters of the black vomit is examined by the author with his customary learning and patient research. He dismisses, as antiquated and untenable, the opinion of its being inspissated or altered bile; and refutes the modern and more accredited belief of its being a morbid secretion from the stomach, to make way for what he avers, and as we cannot but think, to be the true doctrine, viz: that it is the product of hemorrhagic discharge from the blood vessels of the stomach. We wish much that we could introduce his able summary of the arguments in proof of this latter position, that the black vomit "is nothing but blood in a peculiar state of alteration." The characteristic properties of this matter, may, in a great measure, be imitated by the addition of hydrochloric or other acid; and also by mixing the blood of the vena cava with a whitish, acid-smelling liquid, found in the stomach of the same individual, who has died of yellow fever. Direct evidence to the same purport, is furnished by chemical analysis and microscopical examination, in part undertaken at the instance of the author.

Animalculæ of the acarus tribe have been found, by some observers, in the black vomit; but the fact has not been confirmed by the majority of those who have directed their attention to the subject. Different kinds of fungi are evolved from black vomit, under peculiar circumstances.

"The black vomit being recognized to be blood acted upon by the acid contents of the stomach, we have no difficulty in perceiving, that much of the difference it presents, in regard to its physical appearance, will depend on the manner in which the blood is effused into the stomach—whether drop by drop or in a stream—and on the degree of acidity of the gastric secretion, or the quantity of serous fluid it meets in this organ."

The next chapter (XIV) treats of the Tongue, Throat, Respiration and Pain: it is replete with minute specifications of the different symptoms furnished from these sources. The remarks on the tongue are particularly full. Pain, as an almost invariable attendant on the yellow fever, demands, and has received, due attention and notice. That in the head is an early and nearly constant symptom of the disease. "It has been observed, in a greater or less degree, in all epidemics on record, and has attracted the notice of medical inquirers in all climates, and no where more than in this city, in all the sickly seasons of which it has played a conspicuous part in the catalogue of symptoms." Perhaps more frequent and not less severe than headache is the pain in the lumbar regions, in those attacked with yellow fever. The gastric and other abdominal organs, are also the seat of severe suffering in this disease.

Suppression of urine is always of bad augury in yellow fever, and in a large majority cases, in which it occurs, portends a fatal result. "It takes place sometimes, at an early period or during the second stage of the disease. Generally, however, it comes on about the accession of the third."

Among the symptoms drawn from the muscular system, the most surprising, and that which is apt to deceive a person not familiar with the disease, is the retention, or perhaps still oftener, the restoration of muscular strength, so that the patient is able to sit up, walk about the room, or even go down stairs, or into the street as if he were in his usual health. These are what Dr. Caldwell terms, appropriately enough, walking cases. This morbid strength has been retained until within a few minutes of dissolution—a result which it almost always indicates.

The Pathological Anatomy of yellow fever is detailed with great care by the author; but we cannot here venture on even a brief notice of his labors in this way, beyond a few remarks on the appearance of the stomach, as observed after death. "This organ of all others of the body, may justly be regarded as the one most generally and seriously implicated in the yellow fever, and in which the marks of disease are most frequently discovered after death." These marks are described with great minuteness, and their value is tested by the aid of the scalpel and microscope, in the present work, to which the reader must turn for the desired information.

The changes in the appearance and texture of the liver, are dwelt on with some emphasis. Its peculiar color—"light yellow nankeen, fresh butter, straw, coffee and milk, gum yellow, buff, gamboge, light orange, or pistachio"—known and described over and over again, did not fix the attention of medical men, so pointedly as it has done by the announcement and publication of the observations on the subject made by Louis, in the epidemic of Gibraltar, in 1828. Dr. La Roche tells us, as we had learned from different quarters, that this coloration of the liver is far from being a thing of constant occurrence. It has been attributed by Budd and others to fat, far short, however, of the extreme deposit of this substance met with in phthisis.

The chapter on the correspondence between anatomical lesions and symptoms, does not establish any thing conclusive. "In the yellow fever, more perhaps than in any other disease, it is difficult, if not impossible, to connect, as cause and effect, the symptoms observed during life with evidences of textural changes, discovered after death." This position is sustained by passing in review the state of the several organs or apparatuses, whose functions are supposed to be more especially disturbed in the disease.

Critical Days and Critical Efforts constitute the subject of another chapter, in which the author inclines to the affirmative side of the question, and adduces facts to show the benefits following epistaxes, and increased discharges of urine and sweat, which seem to have been critical, as being followed by a marked abatement of disease and beginning return to health.

The type of yellow fever is shown to be, first a continued one, constituting a paroxysm of seventy hours duration,and ending with calm and complete apyrexia, which, sometimes, is the harbinger of of complete recovery. In other cases, after a lull of twelve to thirty-six hours—sometimes a day or two—it is "followed by the more alarming and malignant symptoms which, after some time, gradually abate, and terminate in health; or, as more frequently occurs, assume a more serious aspect, and lead to a fatal termination. This state of remission—the *stadium* of Lining, and the *metaptosis* of Moseley (during which the pulse returns to its natural standard, the tongue, skin, and other surfaces lose their morbid appearances, and most of the other symptoms disappear completely, or in great part,) occurs at the period mentioned with greater or less abruptness. It is never or very seldom followed by a recurrence of febrile excitement." The remission of the fever, assumed to be almost a constant feature, is spoken of by the author, who cites the affirmative opinion of many writers, without himself being of this belief. "So far from it, experience shows that in the large majority of instances—especially those of a severe character, the fever takes on, at once, and preserves the continued type; or if it presents occasional risings and fallings, these modifications are not sufficiently well marked to deserve the appellation of remissions, and still less of intermissions."

We must pass over, with a mere mention of their titles, the successive chapters on Complications of yellow fever, its Duration and in Convalescence and Relapse, nor can we dwell, either by synopsis or extracts, on Prognosis, full of interest and instruction though it be, owing to want of room for the purpose. The same remark will apply to Incubation and the Mortality of Yellow Fever in Philadelphia, the Pathology of the disease and its Diagnosis. Amendment might be made in the order in which some of the subjects have been laid down in the present work. Thus, for instance, Incubation ought to precede the Premonitory Symptoms in description as it does in fact, and Diagnosis include Symptomatology, and precede the Prognosis or Semeiology of the disease. Pathology in its large, comprehensive and olden meaning would include all these several divisions, as well as Morbid Anatomy; since it takes cognizance of the causes, symptoms, signs, seats and effects of diseases. To a consideration of the pathology would succeed that of the treatment of yellow fever; its etiology having, according to this arrangement, been described in the first division of pathology. Dr. La Roche enters on the treatment, after etiology, to which latter belong the topics of contagion, non-contagion and infection. Leaving, for a moment, his wise guidance, we shall first speak of the prominent points of the treatment.

A review of the treatment of yellow fever, from first to last, as so fully and perspicuously laid down in the work before us, confirms our belief that, as yet, no new principle in therapeutics, nor happy use of a remedy, on empirical grounds, has been reached on the occasion. The fact, that the physician has to deal with a synocha or inflammatory fever, which has a tendency, after three days, to decline and end in health, or to take a lull and assume a congestive character, with the accompaniment of gastric and other hemorrhages, does not seem to have suggested any peculiarity of treatment. We have yet to learn, whether there is any defined proportion between the violence of the fever, in its first period, and the occurrence of hemorrhage, or whether a diminution of the morbid excitement, by a reducing treatment, gives any positive assurance of an avoidance or prevention of the hemorhages and black vomit. Up to the present time, the indications are the same as in other fevers, viz : to moderate the inflammatory action or excitement of the first stage, to diminish or remove the associated phlogosis of one or more organs, and to husband the strength or recuperative energies of the system, so as to prevent the prostration and feebleness and other symptoms of an adynamic and typhoid state, and to remove them when present. In yellow, as in other continued and in remittent fevers, it has been made a question, whether it is in the power of medicine to cut short or materially abbreviate the duration of the disease ; and in the first as in the latter case, the great weight of authority, based on experimental observation, is on the negative side. The two extreme views of this subject are equally misleading ; viz : the one, which looks to strangling the disease in its birth, or to control its progress in an arbitrary manner, by therapeutical means of undoubted power and efficacy ; the other, which would only allow us to see an undeviating and uniform march of the disease, made up of excitement followed by remission, of phlogosis by resolution, and of general or local perturbation and derangement by crises, more or less complete, ending, according to the completeness of the crises, either in convalescence or in death. While rejecting extremes, the prudent practitioner will take useful hints from the advocates of each of them; and in his intentness to relieve present fever, pain and other morbid phenomena, he will remember that he has no means, for this purpose, of uniform certainty of operation, at his disposal, none which he can employ with the hope or intention of breaking up the stages of the fever, or of producing evacuations, which shall be preferable to or more certain than those occurring in its progress, and bringing about a crisis of the disease. Hence, he will be continually asking himself: whether a particular symptom, although it is troublesome and even distressing to the patient, should, of necessity, call for the administration of remedies which would probably produce other not pleasant symptoms, and interfere with the progress towards a remission or a crisis?

Assuming that the yellow fever is caused by a poison circulating with the blood, and through it acting deleteriously on the organs and tissues, the great indication for cure would be the use of such remedies as would neutralise this poison, and eliminate it from the system. The theory is plausible, but it lacks something of completeness, by our not having yet ascertained what the poison is, or whether there be any poison at all.

The author of the work before us, shows how carefully he has studied all the essential points, as well as the secondary and collateral ones of treatment, when he says: "The idea of *curing* the disease, or greatly abridging its course, is entitled to little confidence. To nature must be left the chief management of the case; time must be allowed for the elimination of the poison; and the physician must be impressed with the conviction that in cases where no marked organic mischief has been done, or is likely to occur, he must keep his hands off as much as possible, and restrict his agency to the employment of such means as are strictly necessary to meet particular indications."

After remarking on the rapid course of the fever, and the early and sudden appearance of dangerous symptoms, the author counsels continued watchfulness on the part of the physician, and immediate recourse to the remedies which he deems most efficacious for moderating the violence of the onset and preventing the occurrence of secondary evils and complications. The first part of the treatment, or rather the treatment in the first or inflammatory stage, is antiphlogistic. "Of the indispensable necessity of the antiphlogistic and evacuant treatment, which in this, as in other fevers of a kindred nature, consists in sanguine evacuations, sedatives—internal and external—and purgatives, there can be no doubt." The enforcing remark is made that it is only during the first stage, and indeed during the early portion of it, that these remedies can be used with advantage; and the cautionary one, that they are not, as a general rule, to be used with the same freedom as in ordinary inflammations.

The antiphlogistic treatment, beginning with blood letting, is presented with the requisite fulness, and the estimate to be put on each remedy is measured by the testimony of those who on the one hand, have found it serviceable, and who on the other deem it either inefficient or hurtful. Bloodletting has in its favor the greater number of advocates, and is, though not very explicitly, approved of by the author, under specified conditions, and with certain restrictions. Emetics are mentioned, but for the most part to be reprobated, unless there be a full and an overloaded stomach at the beginning of the fever. Purgatives are recommended as undoubtedly serviceable, and the claims of calomel, as the best of this class, and the alleged necessity by its means, of acting on, and of restoring the biliary secretion are investigated. Mercury, in reference to its sialogogue effects and constitutional action, is subjected to the tests of experience, authority and reasoning, and is found to be undeserving of our confidence in the treatment of yellow fever. Diaphoresis is desirable, and alleviates the fever, but most diaphoretics are stimulating and tend to aggravate the symptoms—a distinction this on which the author lays due stress.

Sedatives, anti-emetics and astringents are next spoken of. Blistering figures among the remedies to stop vomiting and calm the stomach. Properly it belongs to the class of counter-irritants. We shall not repeat the means resorted to under the several heads just mentioned, but must refer to the pages of the work itself for abundant therapeutical details on these several topics. So, also, with external applications; prominent among these is cold bathing and affusion, and the use of water by cloths or the wet sheet, and also warm and tepid bathing. Among the most efficacious of all the agents specified in the work, cool or fresh air is entitled to the greatest confidence, acting as it does on two great surfaces ; the cutaneous and the pulmonary.

The author speaks of counter-irritants to the skin under the head of *Counter-Stimulants.* This latter term had better be restricted to the class of remedies thus designated by the Italian school, which regards them as the very opposite to and counter-acting excitement and the effects of stimulants.

*Stimuli* and *tonics* follow in review, and of the latter bark and and sulphate of quinia are brought up, and their appropriate claims examined. Small credit is granted to the latter on the score of its alleged power of cutting short the disease by what has been called the abortive treatment. Abortive it would, indeed, seem to have proved itself. The moral treatment, diet and the treatment of convalescence conclude this part of the subject. They abound in wise observations and available useful advice.

By far the larger part of the second volume, of which the treatment of the fever takes up rather more than a hundred pages, is occupied with the subject of the etiology of the disease, and the dependent questions of contagion, non-contagion and infection. These, alone, would constitute a work of great value to the medical inquirer and philosopher, to whom they supply a body of facts and pregnant suggestions, which are no where else to be found collected and embodied, and which would be thought a rich reward to the solitary student for the labor of many years. The modifications in the human constitution resulting from climate, temperament, age, sex, the operations of the brain and sense, food and drinks, occupations and professions, and all the atmospheric agencies of temperature, light, electricity, pressure, humidity, and humidity and heat, vicissitudes of temperature, and winds are examined; and the actual and relative influence of each modifier is exhibited with abundant illustrations and examples. Over this favorite ground of ours, we should like much to travel with the author, but cannot. Still less are we able, with the printer's caveat against occupying farther space before us, to do common justice to the great theme of the origin and mode of extension of yellow fever. We must content ourselves with saying, in perfect sincerity, that, had a high court of medical judicature, consisting of many of the ablest men in the profession, been established, with power to summon to its aid the most learned of our bibliographers, and the most acute and industrious of our *savants*, the whole question in its several divisions of the foreign or domestic origin of yellow fever, its reputed contagiousness and actual non-contagiousness, were it narrated, discussed and sifted for an entire twelve-month, would not be elucidated by the same amount of learning and positive research that distinguish these labors of Dr. La Roche.

If the advocates of importation and contagion feel that they are overwhelmed by the cogency and weight of the adverse testimony and argument, farther strengthened by the matter of the Appendix, they must, at the same time freely admit, that no where, so well as in these pages, can they marshall for their defense, such an array of plausible statements and ingeniously put co-relations. Few will be found, hereafter, bold enough to incur the imputation of sciolism, by a discussion of the subject, and, above all, by taking side as partisans, without a due knowledge of the contents of the present work.

The means of Prophylaxis, at the close of the second volume, are clearly and distinctly laid down with a due regard to material and tangible poisons and nuisances, and corresponding counteractives and purifiers; but without hypothesis or mystification, and without furnishing an apology for a neglect of active sanitary duties at home, under the pretext of guarding against imaginary evils from abroad, by an expensive, vexatious and inefficient attempt to exclude the fever, by what is generally known as the quarantine system. This topic of prevention is one interesting, in an equal degree, to physicians and medical corporations, boards of health and municipal bodies, and for a clear understanding of it, and impliedly a better knowledge of their duties in the matter, they will, we hope, take the pages of Dr. La Roche for their manual of reference and instruction. We would go farther, and claim for the work a place in the department of general and literary history, and, as such, ask for its being introduced into public and other libraries, with works of this description.

In conclusion, we may here remind our readers of the language which we held in anticipation of the publication of the present work, when the production of the author on Pneumonia, &c. was under review. We said, in reference to his having collected large materials on yellow fever, and our desire to see them arranged and published: "The profession would then, everywhere, be able, with a feeling of just pride, to point to the most learned, comprehensive and accurate work on yellow fever which has ever appeared." Verified as this prediction has been to its full extent, can we be accused of assuming too much, in making ourselves, for the nonce, the exponents of the wishes of our medical brethren that Dr. La Roche would take up the subject of other fevers and treat them with the ability and success so signally displayed in the two works now before the public. Medical literature and medical logic, and the clinics of febrile diseases, would, we are sure, gain by his new labors, as they have gained, beyond all peradventure, by his former ones.

---

*The Practical Anatomist; or, Guide to the Student in the Dissecting Room.* By J. M. Allen, M. D., Professor of Anatomy in Pennsylvania College. Blanchard & Lea. 1854.

There is no book bought by the student which exercises a more important influence upon his character as a student, and, perhaps, as a practitioner, than his Dissector. It depends, in a great measure, upon the impression which this book makes on his mind, whether he will pursue anatomy with pleasure, or, as a mere necessity.

If the volume, placed in his hand to assist him in his unprepossessing labor, be such as to facilitate his studies, one which actually takes the place of the living teacher, showing him how to begin and pursue every subsequent step, then anatomy is pursued intelligently and satisfactorily.

Many of the works which have been sold in this country as Dissectors, have been mere epitomes of large works on Special Anatomy and, in reality, have been of no use to the dissector; on the contrary, they have rather served to confound him, and, perhaps, disgust him with the pursuit.

The sheets of the above named work give us a most favorable idea of its utility and adaptation to the object intended. The student who commences his early dissections with this assistant, will not have to regret the time and labor spent in the dissecting-room.

It is divided into three parts. The first, contains the head and neck; the second, embraces the upper extremities, back and thorax; the third, includes the abdomen and its contents and the lower extremities.

Each part contains a full description of every structure found in the region of the subject under consideration; so that the student will not be under the necessity of consulting different parts of the book, to find a description of what he will meet with in the processes of dissection.

The author has fulfilled the great desideratum in a work of the kind, in presenting everything successively, as nearly as possible, and in the order in which it will appear, to the student as he proceeds, step by step, in his dissection. This work is to teach *how to dissect*, and not merely to give a description of the parts when dissected.

Those portions of the body, a knowledge of which is so essential to the practitioner, and which can be properly studied only in the dissecting room, are treated of with a considerable degree of minuteness. It is evident that the writer has kept in view the importance of anatomy to the physician as well as to the surgeon; and the attention of the student is directed, from time to time, to the practical bearings of a knowledge of the different parts which he is dissecting.

"When the student has become familiar with the appearance and relations of parts, his attention should be directed to the practical application of this knowledge. If he has, for instance, examined the liver *in situ*, and its relations to contiguous parts, he should then study what would be the effect upon these parts when it had increased to two or three times its natural size; or, if an abscess should be formed in it, the different ways in which the pus might find an outlet. There is no place where he can so well appreciate these things as in the dissecting-room, with the subject before him."—*General Remarks.*

We are glad to see that very few arbitrary rules for finding or exposing different organs and parts are introduced. Anatomy is often taught by inches or other measurements, instead of by relation; we have seen a subject triangulated and squared as if a dead body was a field to be surveyed, and not a complex machine, whose individual parts possessed different measurements in every case. The object of the author is to aid the student in becoming a *practical anatomist*, and not one whose knowledge of the body is of merely an arbitrary or mechanical character.

It will be of a convenient form and size for use in the dissecting-room, and besides being illustrated by numerous woodcuts, it is printed in a manner well adapted for the beginner, *i. e.*, it is paragraphed and leaded in such a manner that references are easily made.

Dr. Allen's position as an anatomist, with his experience and reputation in this department of teaching, will place this work far above every American competitor.

---

*A Manual of Pathological Anatomy.* By CARL ROKITANSKY, M. D., Professor in the University of Vienna, etc. Translated from the last German edition by Wm. Edward Swayne, M. D., Edward Sieveking, M. D., Charles Hewitt Moore, George Day, M. D., F. R. S. Four volumes in two. Philad. Blanchard & Lea, 1855.

Few practitioners can be ignorant of the high reputation of Prof. Rokitansky's work on Pathological Anatomy. Its passage through three editions in Germany, taken in connection with the high testimony to its merits by its translation into English under the auspices of the Sydenham Society, evinces the estimation in which it is held throughout Europe. It is everywhere, in fact, acknowledged to be foremost and unrivalled in its department. Only the most earnest professional ardor, the most persevering labor could have enabled its author to amass the immense amount of information contained in its pages. The time and work expended on it may be judged of by the fact, that the number of bodies dissected by him is estimated at 30,000. "As a register of well-observed, well-weighed and well-arranged facts," its re-publication here cannot fail to contribute to the dissemination and advancement of sound pathological knowledge, and should be warmly welcomed by every lover of his profession.

It is impossible for us to present any digest of its contents; our limits allow us only to state, that the 1st volume (published as the last in date, by the Sydenham Society,) treats of general pathological anatomy, under the heads of anomalies, in respect of the number, size, form, position, connection, color and consistence of parts; separations of continuity, and anomalies of texture and contents; that the 2d, discusses the abnormities of the digestive apparatus, of the urinary organs and of the sexual organs; the 3d, of the anomalies and diseases of the cellular tissue; of serous and synovial membranes; of mucous membranes; of anomalies and diseases of the skin; of the fibrous system; of the osseous system; of cartilages; of the muscular system, and of the nervous system; and that the 4th, treats of abnormal condition of the respiratory organs, and of the organs of circulation.

With the following remarks, from a notice of the work in the Boston Medical and Surgical Journal, we entirely agree. "We need, therefore, say no more in order to recommend it to the attention of the profession, who are under the greatest obligations to the Sydenham Society, for the admirable translation which it has given to the English public, at a heavy expense and without any adequate return. We feel bound to say that the publishers of the American edition, in appropriating the translation of the Sydendam Society, ought, at least, to have acknowledged, on the title page, the source from whence it was obtained; and, indeed, all such reprints, unless authorized by their rightful proprietors, are, in our opinion, wholly unjustifiable."

We strongly recommend our readers to obtain a copy of Dr. Rokitansky's work. No library should be without it.

---

*Transactions of the State Medical Society of the State of New York.* Albany, 1855.

We have read with considerable care and interest, the "Report on Dislocations, with especial reference to their results," by Dr. Frank H. Hamilton, of Buffalo. In this paper, Dr. H. has evinced the same careful and investigating spirit that has characterized his inquiries concerning fractures. There is an effort made to discover how far these injuries are followed by perfect cures, or rather, we should say, by imperfect cures, "of the amount and character of the maiming which is likely to ensue where the reduction has been effected, and of the length of time during which such maiming may be reasonably expected to continue." He has collected the cases coming under his own experience principally as consulting surgeon, written them out at length and tabulated them with the results. He enumerates nine cases of the clavicle; forty-four cases of dislocation of the humerus; fourteen of the radius; twenty-one of the radius and ulna; fourteen of the metacarpus and fingers; seven of the femur; seventeen of the tibia; four of the tibia and fibula; and two of the tarsal bones.

The amount of material collected is certainly creditable to the industry of the author, and of the value of such contributions no well informed practitioner can doubt.

The table of contents of the Transactions for this year embraces twenty-one articles, many of which can be read with profit by the profession generally.

---

*The Cause and Prevention of Yellow Fever, contained in the Report of the Sanitary Commission of New Orleans.* By E. H. Barton, M. D. Chairman of the Sanitary Commission, &c. Philadelphia, Lindsay & Blakiston.

Our May number contained a full notice of the "Report of the Sanitary Commission of New Orleans, of which Dr. Barton's paper was the largest part. As the edition was soon exhausted, Dr. Barton, to supply the demand, at home and abroad, has had his portion of the work re-published, with considerable additions; the most material of which, is a paper read by him before the "Academy of Sciences," defending his opinions and further explaining the principles contained in his report. We take this opportunity of repeating our high opinion of its merits.

